# Characteristics of Hypoglycemic Diabetic Patients Visiting the Emergency Room

**DOI:** 10.1155/2020/3612607

**Published:** 2020-07-20

**Authors:** Tong Min Kim, Hyunah Kim, Seung-Hwan Lee, Jae-Hyoung Cho, Hyunyong Lee, Hyeon Woo Yim, Kun-Ho Yoon, Hun-Sung Kim

**Affiliations:** ^1^Department of Medical Informatics, College of Medicine, The Catholic University of Korea, Seoul, Republic of Korea; ^2^College of Pharmacy, Sookmyung Women's University, Seoul, Republic of Korea; ^3^Division of Endocrinology and Metabolism, Department of Internal Medicine, Seoul St. Mary's Hospital, College of Medicine, The Catholic University of Korea, Seoul, Republic of Korea; ^4^Clinical Research Coordinating Center, Catholic Medical Center, The Catholic University of Korea, Republic of Korea; ^5^Department of Preventive Medicine, College of Medicine, The Catholic University of Korea, Republic of Korea

## Abstract

**Introduction:**

Severe hypoglycemia can be life-threatening; therefore, it is important to identify the characteristics of the hypoglycemic patients. The aim of this study is to analyze the type and characteristics of diabetic patients with hypoglycemia who visited an emergency room.

**Methods:**

We included diabetic patients with hypoglycemia who visited the emergency room of St. Mary's Hospital in Seoul from January 2009 to August 2018 in the study. Hypo_S group patients visited the emergency room once whereas Hypo_M group patients visited twice or more. We also compared the incidence of cardiovascular disease between the groups within 5 years after hypoglycemia.

**Results:**

A total of 843 patients were included in this study, with a mean age of 71 ± 14 years and average glycated hemoglobin (HbA1c) level of 6.7 ± 1.4%. For patients with hypoglycemia, lower body mass index, lower HbA1c, shorter diabetes duration, and lower glomerular filtration rate have a statistically significant relationship with patient characteristics in the emergency room group (all *p* < 0.001). Hypoglycemia symptoms were most frequently observed between 6:00 and 12:00 am (*p* < 0.001). Cardiovascular diseases within 5 years after discharge were more frequent in the Hypo_S group than in the Hypo_M group; however, there was no statistical significance. The frequency of aneurysms was significantly higher in patients with hypoglycemia than in other patients in the emergency room (*p* < 0.05).

**Conclusion:**

Relatively thin older patients with a diabetes duration shorter than 10 years and good blood sugar control showed higher frequency of visits to the emergency room due to hypoglycemia. For these patients, medical staff should always be mindful of their susceptibility to hypoglycemia when prescribing insulin or OHA and educate them on the prevention of hypoglycemia.

## 1. Introduction

Hypoglycemia is defined as a decrease in blood glucose levels below 70 mg/dL in patients with diabetes treated with insulin or oral hypoglycemic agents (OHAs) [1]. Clinically significant hypoglycemia is defined as blood glucose less than 54 mg/dL, and severe hypoglycemia is defined as a hypoglycemic condition requiring the help of other agents for recovery. If left untreated immediately, the possibility of decreased cognitive function, dementia, arrhythmia, coronary artery disease, and mortality increases. It is important to prevent hypoglycemia in advance because patients with hypoglycemia in the emergency room are more likely to have hyperglycemia [2], and doctors should educate patients who are at high risk for hypoglycemia. Therefore, it is also important for healthcare providers who prescribe insulin or OHA to identify the characteristics of the hypoglycemic patients who they want to have in the emergency department.

Three intensive randomized clinical trials, namely, Action to Control Cardiovascular Risk in Diabetes (ACCORD) [3], Action in Diabetes and Vascular Disease—Preterax and Diamicron Modified Release Controlled Evaluation (ADVANCE) [4], and the Veterans Affairs Diabetes Trial (VADT) [5], showed that intensive glycemic control, aiming at HbA1c level of <6.0%–6.5%, tends to increase the risk of hypoglycemia and mortality. The medical staff should know in advance which patients are at high risk for hypoglycemia, and thus, they can elevate their target blood glucose levels. If medical staff can determine the characteristics of patients at high risk for hypoglycemia beforehand, it will be very helpful for the treatment of diabetes and the choice of an individualized diabetic agent will be possible.

Many people who have experienced hypoglycemia have had multiple hypoglycemic episodes even though they should have been careful about recurrence of hypoglycemia [6]. In this study, we aimed to analyze the type and characteristics of diabetic patients who visited an emergency room with hypoglycemia. The purpose of this study was to determine the difference in risk factors according to the occurrence of hypoglycemia by examining characteristics of individual patients according to the number of visits to the emergency room, medication dose, and presence or absence of various diseases. Subgroup analysis was conducted to determine whether there were any differences in the number of visits to the emergency room due to hypoglycemia and the incidence of various cardiovascular events occurring within the next 5 years.

## 2. Methods

### 2.1. Population

This study was a retrospective study using electronic medical records of Seoul St. Mary's Hospital, the Catholic University of Korea, Seoul, Korea. From January 1, 2009, to December 31, 2017, patients with hypoglycemia who visited the emergency room of Seoul St. Mary's Hospital over this 9-year period were included. Those diagnosed with diabetes and aged over 18 years who were prescribed an oral hypoglycemic agent (OHA) or insulin were selected. Patients were categorized into two groups based on the number of visits to the emergency room: those with a single visit to the emergency room were categorized into the Hypo_S group and those with multiple visits (twice or more) into the Hypo_M group.

### 2.2. Study Design

Hypoglycemia was defined using the International Classification of Diseases ICD10 classification: ICD10 classification E162, E1063, E1163, E1263, E1363, E1463, E160, and E161. We hypothesized that hypoglycemia due to a drug (or insulin) was not the cause of diabetes mellitus (DM), and we excluded it from this study. We extracted basic information such as date of birth, height, body mass index (BMI), systolic blood pressure, diastolic blood pressure, and number of patients who visited the emergency room with hypoglycemia. In accordance with NIH [7], patients' alcohol consumption termed as “binge” was defined as more than one can of beer, more than one glass of wine, and more than two glasses of Korean traditional alcohol, *Soju*.

All OHAs or insulins that were taken within 3 months before the emergency room visit were examined, including details such as the names of all medicines not related to diabetes, the number of prescriptions, and details on the prescriptions. In the case of the first visit to the hospital, the drug was prepared by direct chart review. Blood tests were performed on patients who were hospitalized. Information was extracted for the following blood tests: fasting glucose, HbA1c, insulin, C-peptide, blood urea nitrogen (BUN), creatinine, glomerular filtration rate (GFR), total protein, albumin, sodium, potassium, total bilirubin, aspartate aminotransferase (AST), alanine aminotransferase (ALT), creatine phosphokinase (CPK), lactate dehydrogenase (LDH), amylase, lipase, prothrombin time, erythrocyte sedimentation rate (ESR), high-sensitivity C-reactive protein (hsCRP), white blood cell (WBC) count, absolute neutrophil count (ANC), red blood cell (RBC) count, platelet count, hemoglobin, hematocrit, alkaline phosphatase (ALP), gamma-glutamyl transpeptidase (rGTP), total cholesterol (TC), triglyceride (TG), high-density lipoprotein (HDL), low-density lipoprotein (LDL), and osmolality.

ICD10 classification was also used to determine the presence or absence of accompanying diseases: I20-25 (ischemic heart diseases (IHD)), I60-69 (cerebrovascular diseases (CVD)), I10-15 (hypertensive diseases), I71-73 (aneurysm dissection), heart failure (I50, I11.0, I13.0, I24.8), N179 (renal failure), I46 (sudden cardiac death), and C (cancer).

### 2.3. Subgroup Analysis of Various Cardiovascular Events

From January 2009 to June 2013, patients who visited the emergency room over 4 years and 6 months were selected to determine the incidence of cardiovascular disease within 5 years after discharge from the emergency room. The cardiovascular diseases investigated were IHD, CVD, hypertensive diseases, heart failure, renal failure, cancer, and sudden death. The ICD10 classification was also used.

### 2.4. Chart Review and Data Quality Management

A direct chart review was conducted to determine whether diabetes patients visited the emergency room purely for hypoglycemia or hypoglycemia after admission for other reasons. The chart review was used to check the time at which hypoglycemia first occurred and simultaneously confirm the time of emergency room visit.

### 2.5. Privacy Protection

When extracting the relevant clinical research data, we did not include personal identification information (name, resident registration number, etc.), basic information (anonymized patient number, date of birth, sex, details of medications such as prescription name, prescription drug code, product name, ingredient name, dosage, and number of days prescribed), and diagnosis information (diagnosis name and diagnosis code, presence/absence of diagnosis name, and initial diagnosis date). Only laboratory testing information (test prescription code, test prescription date and time, test date, test result, abnormality, and reference upper/lower limit) and drug evaluation information (drug maintenance, interruption/change) were collected. Patient registration numbers were obtained after anonymization at data collection.

### 2.6. Statistical Analysis

Baseline variables were expressed as means and standard deviation or median and interquartile range for continuous variables and numbers including percentages for categorical variables. Hypoglycemia status was compared using independent *t*-test or Wilcoxon rank sum test for continuous variables and chi-square or Fisher's exact test for categorical variables. Difference in incidence ratio was evaluated by survival analysis using log-rank test to compare hypoglycemic admission status and various disease entities. All statistical analyses were performed using SAS version 9.4 (SAS Institute, NC, Cary), and a *p* value below 0.05 was considered statistically significant.

## 3. Results

**Figure 1:**
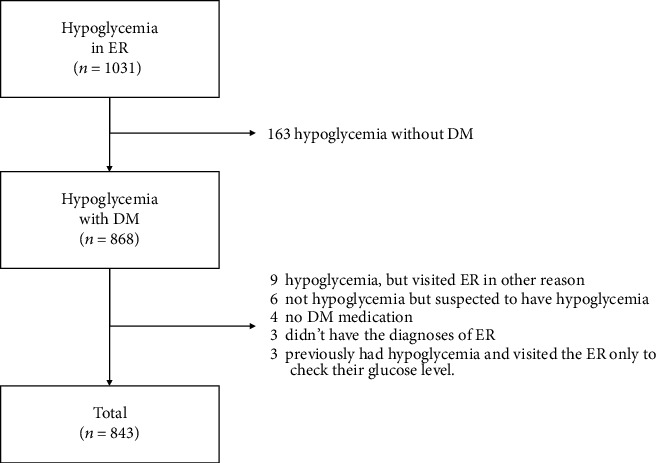
Flowchart of patient enrollment and status (*n* = 843). DM: diabetes mellitus; ER: emergency room.

From January 1, 2009, to December 31, 2017, a total of 1,031 patients with hypoglycemia were admitted to the emergency room of the Catholic University of Korea. Of these, 163 did not have diabetes and were excluded (Figure 1). Exclusions were also due to the following: nine patients who had hypoglycemia but visited the emergency room not by hypoglycemia, six patients who had no hypoglycemia but suspected to have hypoglycemia, four patients who had diabetes without medicine intake, three patients who did not have the diagnoses of the emergency room, and three patients who previously had hypoglycemia and visited the emergency room only to check their glucose level. Thus, a total of 843 patients were included in the study.

### 3.1. Characteristics of the Hypoglycemic Patients Visiting the ER

The mean age of the patients who visited the emergency room with hypoglycemia was 70 ± 14 years (Table 1). There were 54% (453/843) males and 46% (390/843) females in the emergency room with hypoglycemia; however, the difference was not statistically significant (*p* = 0.548). Among all the patients with hypoglycemia, 2.3% (19/843) were diagnosed with type 1 diabetes, and 97.4% (824/843) were diagnosed with type 2 diabetes.

**Figure 2:**
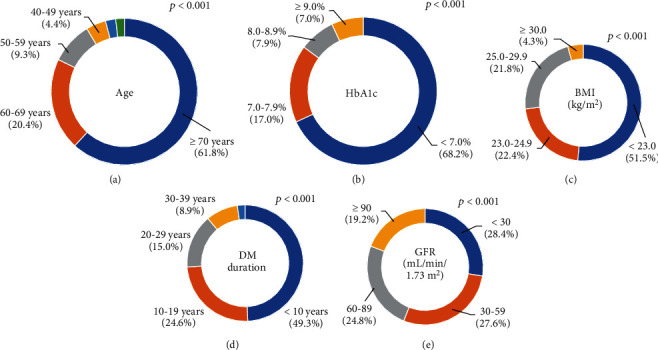
Characteristics of patients who visited the emergency room with hypoglycemia: (a) age, (b) HbA1c, (c) BMI, (d) duration of diabetes, and (e) GFR. BMI: body mass index; DM: diabetes mellitus; GFR: glomerular filtration rate.

Among them, 61.8% (521/843 patients) were over 70 years of age, 20.4% (172/843) were aged 60-69 years. The number of patients diagnosed with hypoglycemia in the emergency room increased significantly with age (*p* < 0.001) (Figure 2(a)). Due to some missing HbA1c at ER visit data, we collected 559 HbA1c at ER visit data from 843 patients. The mean HbA1c of the patients who visited the emergency room with hypoglycemia was 6.7 ± 1.4%. Most patients (68.2%, 381/559) had an HbA1c level of less than 7%, 17.0% (95/559) had levels 7.0–7.9%, 7.9% (44/559) had levels 8.0–8.9%, and 7% (39/559) had levels more than or equal to 9.0% (*p* < 0.001) (Figure 2(b)). Due to some missing weight and height data, we collected 491 BMI data from 843 patients. The mean BMI of patients with hypoglycemia was 23.0 ± 3.7 kg/m^2^; 51.5% (253/491) had a BMI of less than 23 kg/m^2^. The proportion of patients visiting the emergency room was significantly higher in those with lower BMI (Figure 2(c)).

Due to some missing DM duration data, we collected 280 DM duration data from 843 patients. The mean duration of DM in patients who visited the emergency room with hypoglycemia was 12 ± 11 years. Among them, 49.3% (138/280) had diabetes for less than 10 years. In the second group, 24.6% (69/280) had diabetes for the duration of 10–19 years, 15.0% (42/280) for 20–29 years, and 8.9% (25/280) patients for between 30 and 39 years. Finally, the longer the duration of the DM, the shorter the duration of the disease (*p* < 0.001) and the longer the duration of DM (2.1%, 6/280) (Figure 2(d)). The mean GFR of patients who visited the emergency room with hypoglycemia was 57 ± 38 mL/min/1.73 m^2^. Due to some missing GFR data, we collected 791 GFR data from 843 patients. The highest GFR of 30–59 mL/min/1.73m^2^ was seen in 28.4% (225/791) patients, whereas 27.6% (218/791) patients had a GFR of less than 30 mL/min/1.73m^2^. The number of patients with lower GFR among those visiting the emergency room with hypoglycemia was significantly higher than the number of patients with higher GFR (*p* < 0.001) (Figure 2(e)).

Almost half (48.0%, 405/843) of the patients controlled their blood glucose using insulin. Among the oral hypoglycemic agents, usage of sulfonylurea (SU) was the most common, being used by 34.4% (290/843) of patients, followed by metformin by 19.6% (16/843), dipeptidyl peptidase-4 inhibitor (DPP4i) by 16.5% (139/843), meglitinide by 5.2% (44/843), thiazolidinediones by 2.4% (20/843), acarbose by 1.0% (8/843), and sodium-glucose cotransporter 2 inhibitors (SGLT2i) by 0.2% (2/843) of patients (Table 1). With regard to accompanying disease, the incidence of ischemic heart disease was 26.0% (219/843), followed by cerebrovascular disease (24.0%, 202/843), hypertensive disease (22.0%, 185/843), and aneurysm (18.9%, 159/843). Cancer had been already diagnosed in 15.4% (13/842) of the patients.

### 3.2. Hypoglycemic-Related Characteristics of Patients Who Visited the Emergency Room with Hypoglycemia

**Figure 3:**
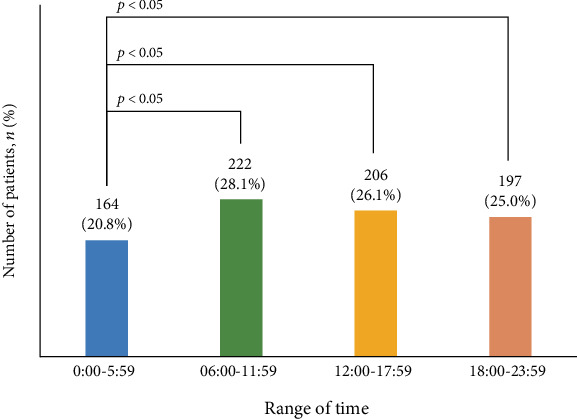
Hypoglycemia-related characteristics of patients who visited the emergency room with hypoglycemia.

Due to some missing blood glucose data, we collected 515 blood glucose data from 843 patients. The average prehospital blood glucose level of the patients who visited the emergency room with hypoglycemia was 37.0 ± 13.0 mg/dL. Most patients (32.8%, 169/515) had blood glucose levels of 30–39 mg/dL before admission. Blood glucose levels of 20–29 mg/dL and 40–49 mg/dL before visits to the emergency room were found most frequently in 20.8% (107/515) and 21.7% (112/515) of the patients, respectively. The most clinically significant hypoglycemia was found in 11.5% (59/515) of the patients with blood glucose level of 50 mg/dL. Due to some missing the time of symptom occurrence data, we collected 789 time of symptom occurrence data from 843 patients. Before the emergency room visit, hypoglycemic symptoms occurred most frequently in the period from 6:00 am to 11:59 am in 28.1% (222/789) of the patients, followed by 26.1% (206/789) of the patients from 12:00 to 17:59 and 25.0% (197/789) from 18:00 to 23:59. From 00:00 am to 5:59 am, 20.8% (164/789) of the patients had the least number of hypoglycemic symptoms, and statistically significant differences were observed (Figure 3).

### 3.3. Patient Characteristics according to the Frequency of Visits to the Emergency Room with Hypoglycemia

Eighty-eight percent (744/843) of the patients visited the emergency room with hypoglycemia and 12% (99/843) of the patients visited the emergency room twice (*p* < 0.001) (Table 2). The mean age of the Hypo_S group patients was significantly higher than that of the Hypo_M group patients (*p* = 0.365). The BMI was not significantly different between the Hypo_S and Hypo_M groups (*p* = 0.346). Type 1 DM was more prevalent in the Hypo_M group than in the Hypo_S group (16 vs. 3); however, there were no significant differences (*p* = 0.480). Similarly, there were no significant differences in DM duration between the two groups (8.0 vs. 10.0 years, *p* = 0.573).

Insulin was administered in half of the patients in both groups (48.1% vs. 47.5%, *p* = 0.904); however, there was no significant difference according to the number of visits to the emergency room. Sulfonylurea (33.7% vs. 39.4%, *p* = 0.266) and meglitinide (5.4% vs. 4.0%, *p* = 0.575) did not show any significant differences between the two groups. The values of HbA1c at the time of admission to the emergency room were 6.7 ± 1.4% in the Hypo_S group and 6.7 ± 1.3% in the Hypo_M group; however, there was no significant difference between the two groups (*p* = 0.667). There were no clinically significant differences between the two groups regarding creatinine (1.14 vs. 1.22 mg/dL, *p* = 0.505), estimated GFR (55.0 vs. 51.0 mL/min/1.73m^2^, *p* = 0.763), hemoglobin (11.6 ± 2.1 vs. 11.87 ± 1.84 g/dL, *p* = 0.224), hematocrit (34.4 ± 5.9 vs. 35.2 ± 5.1 M/*μ*L, *p* = 0.188), osmolality (294 ± 16 vs. 292 ± 18 mOsm/kg, *p* = 0.259), and other blood tests. In regard to total bilirubin (0.4 vs. 0.0 mg/dL, *p* = 0.020), AST (27 vs. 25 IU/L, *p* = 0.018), and prothrombin time international normalized ratio (PT_INR) (1.13 ± 0.42 INR vs. 1.03 ± 0.09, *p* < 0.001), the Hypo_M group had a significantly lower value than the Hypo_S group. However, the platelet count in the Hypo_M group was significantly higher than that in the Hypo_S group (244 ± 75 vs. 221 ± 93 K/*μ*L, *p* = 0.008).

In patients with a history of illness, the incidence of heart failure (7.1% vs. 4.0%, *p* = 0.251) and IHD (26.1% vs. 25.3%, *p* = 0.861) was higher in the Hypo_S group than in the Hypo_M group. Hypertensive disease (23.2% vs. 21.8%, *p* = 0.742), cerebrovascular disease (24.2% vs. 23.9%, *p* = 0.945), and aneurysm (23.2% vs. 18.3%) were found to be higher in the Hypo_M group than in the Hypo_S group. In the case of cancer, the Hypo_S group showed significantly higher value than the Hypo_M group (16.7% vs. 6.1%, *p* = 0.006).

### 3.4. Subgroup Analysis of Cardiovascular Disease Incidence within 5 Years after Discharge (Table 3)

There was a higher incidence within 5 years in the Hypo_S group than in the Hypo_M group with respect to heart failure (5.1% vs. 8.7%, *p* = 0.256), sudden cardiac death (2.2% vs. 2.7%, *p* = 0.678), IHD (15.1% vs. 16.1%, *p* = 0.851), cerebrovascular disease (12.1% vs. 13.0%, *p* = 0.860), hypertensive disease (8.2% vs. 8.8%, *p* = 0.799), renal failure (3.5% vs. 6.9%, *p* = 0.186), and cancer (6.9% vs. 8.5%, *p* = 0.634). However, there were no statistically significant differences between the two groups. Aneurysm, however, showed a statistically higher incidence in the Hypo_M group than in the Hypo_S group (26.8% vs. 14.7%, *p* = 0.022), respectively.

## 4. Discussion

Hypoglycemia is a side effect that is especially likely to occur in diabetics who control blood sugar with oral hypoglycemic agents or insulin [8]. Trauma resulting from hypoglycemia also ranges from mild abrasions to fractures, dislocations, and head injuries. Hypoglycemia is more serious when it results in a visit to the emergency room, and severe hypoglycemia can lead to loss of cognitive ability and dementia [9–13]. In the present study, the mean blood glucose level of patients in the emergency room was 34 ± 14 mg/dL, which met the criteria for severe hypoglycemia.

In this study, the proportion of patients on insulin treatment was almost half at 48.0%. The percentage of patients on insulin treatment in Korea declined to 9.0% in 2016. Nonetheless, half of the patients who wanted to go to the emergency room with hypoglycemia were those treated with insulin. In other words, even if only hypoglycemic training is administered to only 9% of the patients who are treated with insulin, half of the hypoglycemic patients who want to enter the emergency room will be prevented. In addition, the rate of using OH, which is the most common cause of hypoglycemia, was 34.4%, accounting for the largest percentage of the drugs. It is well known that insulin and SU are the most common causes of hypoglycemia [14]. As SGLT2i or DPP4i, which are not known to cause hypoglycemia in recent years, have started to replace SU, the prescription of SU is decreasing. Therefore, hypoglycemia due to SU is expected to decrease gradually. In the future, education about patients who are prescribed insulin may become more important.

In addition to insulin and OHA, this study showed that DM duration was significantly shorter in patients with DM duration of less than 10 years, relatively older age, lower BMI, and HbA1c already reaching the target blood glucose level. This is largely consistent with those demonstrated in other previous studies [1, 2]. In this study, mean duration of DM in diabetic patients was less than 10 years, and the frequency of hypoglycemia decreased as the duration of diabetes increased. In one study, the longer the duration of the DM, the lower the number of patients visiting the emergency room due to hypoglycemia [2]. The longer the DM duration, the more experience of coping with hypoglycemia and the fewer number of visits to the emergency room because of the possibility of self-regulation. In other studies, both in Korea and abroad, the mean duration of diabetes in hypoglycemic patients was 12.7 years, which was not significantly different from the results of this study [2, 15].

The mean age of the patients in our emergency room was 70 ± 14 years and 60% were over 70 years old. When we examine the current status of diabetes in Korea [16], the proportion of diabetics over 70 years old is approximately 30%. Nonetheless, a high percentage (60%) of patients aged >70 years with severe hypoglycemia rather than mere hypoglycemia in this study is seriously concerning. In fact, it is well known that hypoglycemia occurs more frequently in older patients [17]. In many countries, the guidelines already recommend a higher blood glucose target for seniors. In the current Korean Diabetes Association guideline, the target of glycemic control in patients with type 2 diabetes is <6.5% of HbA1c. However, the guideline permits individualization of the goal to prevent the risk of developing hypoglycemia, resulting in short life expectancy, progressive microvascular and macrovascular complications. Closer monitoring of occurrence of hypoglycemia as well as incidence of cardiovascular disease in older patients and in the younger population is required, as there is a higher probability of death if there is no immediate response [8, 9, 12, 13, 18].

In the present study, the mean HbA1c level of patients who visited the emergency room with hypoglycemia was 6.7 ± 1.4%, and a level of less than 7.0% was experienced by 68.2% of patients. In Korea, the HbA1c level in patients with DM [16] is reported to be <7.0% in half of the patients. In other words, about 68.2% of the patients have been admitted to the emergency room with hypoglycemia. Although severe glucose control in diabetic patients may help reduce the incidence of diabetic complications, ACCORD [3], ADVANCE [4], and VADT [5] clinical trials showed that active intensive blood sugar management significantly increased the risk of hypoglycemia and other serious risks, rather than preventing complications. Therefore, for patients who are at risk of hypoglycemia or those who have experienced hypoglycemia at least once, it is necessary to consider the clinical history of the patient and adjust the blood glucose target accordingly.

Low BMI is already well known as a risk factor for hypoglycemia. Among the diabetic patients in Korea, only 25.1% have a BMI of less than 23. However, in this study, more than half (51.5%) of the patients had a BMI of less than 23. In other words, patients who are older and those who experience symptoms of dryness will need to be more cautious about diabetes medication. Baseline GFR in patients is also an important risk factor.

In our previous study [19], we concluded that GFR values were not related to hypoglycemia. In this study, GFR is also thought to be unrelated to hypoglycemia, as there was no difference in the number of patients among the various ranges.

Before visiting the emergency room, symptoms were most frequently experienced between 06:00 am and 11:59 am, with the least number between 12:00 am and 05:59 am. In fact, more than half of severe hypoglycemia occurs at night. This is because of the increase in insulin secretion at dawn. This is probably due to the fact that the patient feels no symptoms of hypoglycemia while sleeping and feels symptoms after waking and visits the emergency room. It is helpful for patients at high risk for hypoglycemia to check their blood glucose before sleeping; thus, the guardian will know the condition of the patient after waking up due to hypoglycemia-induced hypersensitivity.

This study did not reveal important statistical differences between the Hypo_S and Hypo_M groups, i.e., it did not uncover the risk factors in patients for the number of visits to the emergency room. The Hypo_M group showed lower tendency of obesity, binge drinking, and type 1 DM than the Hypo_S group. Total bilirubin, AST, PT (INR), and platelet counts showed significant differences in both groups; however, it was difficult to obtain clinical significance. The Hypo_S group was significantly more frequent compared to the Hypo_M group. The incidence of hypoglycemia was relatively low because of the high prevalence of steroid therapy in patients with cancer and the expected increase in blood sugar due to steroids. In addition, the frequency of hypoglycemia due to hospitalization was considered to be low.

Hypoglycemia was associated with increased cardiovascular disease, hospitalization rate, and mortality [20–23]. However, in this study, the incidence of IHD, CVD, hypertensive disease, heart failure, and sudden cardiac death showed no significant difference. However, we found that the higher the number of visits to the emergency room, the higher the incidence of diseases. This may be due to the fact that the sample consisted of less than 500 patients over 4 years and 6 months, and the incidence was low. However, as there is a tendency for differences in the incidence, it is expected that there will be a meaningful difference between the two groups if the number of subjects is increased over time and if they are observed for longer than 5 years. Additional research is needed to confirm this.

This study presents evidence from research conducted among patients in a real-world setting. However, there were some limitations associated with this study. First, the small sample size and short follow-up duration detract the potential for substantial, unambiguous conclusions, and a meaningful intergroup difference was not obtained. A longer follow-up period is required to obtain meaningful results. Second, the laboratory data comprised objective numerical values. However, patient information including alcohol consumption, height, weight, blood glucose level at the time of hypoglycemia, and the time of hypoglycemia was recorded by medical staff based on the patient responses to direct questions; this may have differed from the actual values because of subjective responses and recall bias.

In conclusion, prevention of hypoglycemia is possible by the early detection of high-risk patients and actively providing them with medical advice by physicians. Increasing target blood sugar levels in elderly patients is already well known. However, relatively thin elderly patients with DM duration of less than 10 years and HbA1c that had already reached target blood glucose need more specific, persistent, and thorough education and attention. Finally, large-scale randomized controlled trials should be undertaken to draw clear conclusions.

## Figures and Tables

**Table 1:** Characteristics of the enrolled diabetic hypoglycemic patients.

Total	
Number, *n*	843
Age (year)	70 ± 14
Female, *n* (%)	390 (46.3%)
BMI (kg/m^2^)	23.0 ± 3.7
Alcohol (binge), *n* (%)	88 (10.4%)
Type 1 DM, *n* (%)	19 (2.3%)
DM duration (year)	4 (1-11)
Medication, *n* (%)	
Insulin	405 (48.0%)
Sulfonylurea	290 (34.4%)
Meglitinide	44 (5.2%)
Metformin	165 (19.6%)
DPP4i	139 (16.5%)
SGLT2i	2 (0.2%)
Thiazolidinediones	20 (2.4%)
*α*-Glucosidase inhibitors	8 (1.0%)
Glucose (mg/dL)	37 ± 13
HbA1c (%)	6.7 ± 1.4
BUN (mg/dL)	24 (16-37)
Creatinine (mg/dL)	1.2 (0.81-2.19)
GFR (mL/min/1.73m^2^)	55.0 (28-80)
Comorbidity, *n* (%)	
Ischemic heart disease	219 (26.0%)
Cerebrovascular disease	202 (24.0%)
Hypertensive disease	185 (22.0%)
Aneurysm	159 (18.9%)
Heart failure	57 (6.8%)
Renal failure	17 (2.0%)
Sudden cardiac death	1 (0.1%)
Cancer	130 (15.4%)

BMI: body mass index; BUN: blood urea nitrogen; DM: diabetes mellitus; DPP4i: dipeptidyl peptidase-4 inhibitor; SGLT2i: sodium-glucose cotransporter 2 inhibitors; GFR: glomerular filtration rate.

**Table 2:** Baseline comparisons between frequencies of visit.

	HYPO_S group	HYPO_M group	*p* value
Number, *n* (%)	744 (88%)	99 (12%)	
Age (year)	70 ± 14	69 ± 15	0.365
Female, *n* (%)	347 (46.6)	43 (43.4)	0.548
BMI (kg/m^2^)	23.0 ± 3.7	22.5 ± 3.7	0.346
Alcohol (binge), *n* (%)	73 (9.8)	15 (15.2)	0.103
Type 1 DM, *n* (%)	16 (2.2)	3 (3.0)	0.480
DM duration (year)	8.0 (4-20)	10.0 (2-20)	0.573
Medication, *n* (%)			
Insulin	358 (48.1)	47 (47.5)	0.904
Sulfonylurea	251 (33.7)	39 (39.4)	0.266
Meglitinide	40 (5.4)	4 (4.0)	0.575
Metformin	149 (20.0)	16 (16.2)	0.363
DPP4i	128 (17.2)	11 (11.1)	0.125
SGLT2i	2 (0.3)	0 (0.0)	>0.999
Thiazolidinediones	15 (2.0)	5 (5.1)	0.074
*α*-Glucosidase inhibitors	7 (0.9)	1 (1.0)	>0.999
HbA1c (%)	6.7 ± 1.4	6.7 ± 1.3	0.667
BUN (mg/dL)	24 (16-37)	23 (16-33)	0.525
Cr (mg/dL)	1.14 (0.80-2.23)	1.22 (0.88-1.92)	0.505
eGFR (mL/min/1.73m^2^)	55 (27-81)	51 (32-75)	0.763
Total protein (g/dL)	6.53 ± 0.81	6.62 ± 0.67	0.255
Albumin (g/dL)	3.63 ± 0.65	3.72 ± 0.59	0.186
Sodium (mmol/L)	138.25 ± 5.25	138.63 ± 4.54	0.499
Potassium (mmol/L)	4.25 ± 0.79	4.17 ± 0.61	0.289
Cl (mmol/L)	102.60 ± 5.88	103.63 ± 4.80	0.058
Total bilirubin (mg/dL)	0.4 (0.31-0.63)	0.4 (0.28-0.54)	0.020
AST (IU/L)	27 (21-39)	25 (20-30)	0.018
ALT (IU/L)	19 (14-29)	18 (13-26)	0.092
CPK, *n*	88 (52-165)	89 (50-179)	0.905
LDH, *n*	497 (411-622)	457 (391-553)	0.006
Amylase (*μ*L/dL)	98 (69-135)	106 (73-144)	0.242
Lipase (U/L)	23 (12-47)	28 (10-52)	0.808
PT_INR (INR)	1.13 ± 0.42	1.03 ± 0.09	<0.001
hsCRP (mg/dL)	0.24 (0.06-1.63)	0.15 (0.04-1.03)	0.062
Platelet count (K/*μ*L)	221 ± 93	244 ± 75	0.008
WBC count, *n*	8.0 (6-11)	8.0 (6-11)	0.502
ANC, *n*	7.16 ± 4.06	6.56 ± 3.55	0.169
RBC count, *n*	3.72 (3.32-4.23)	3.85 (3.50-4.10)	0.215
Hemoglobin (g/dL)	11.6 ± 2.1	11.87 ± 1.84	0.224
Hematocrit (M/*μ*L)	34.4 ± 5.9	35.2 ± 5.1	0.188
ALP (IU/L)	73 (55-123)	60 (45-89)	0.059
Gamma-GTP (IU/L)	31 (17-110)	26 (17-37)	0.086
TC (mg/dL)	144.93 ± 45.88	157.50 ± 18.38	0.519
TG (mg/dL)	73 (53-98)	86 (56-136)	0.557
HDL (mg/dL)	39.39 ± 15.19	49.20 ± 9.26	0.180
LDL (mg/dL)	87.78 ± 41.74	80.67 ± 19.50	0.776
Osmolality (mOsm/kg)	295 ± 16	292 ± 18	0.259
Comorbidity, *n* (%)			
Ischemic heart disease	194 (26.1)	25 (25.3)	0.861
Cerebrovascular disease	178 (23.9)	24 (24.2)	0.945
Hypertensive disease	162 (21.8)	23 (23.2)	0.742
Aneurysm	136 (18.3)	23 (23.2)	0.237
Heart failure	53 (7.1)	4 (4.0)	0.251
Renal failure	15 (2.0)	2 (2.0)	>0.999
Sudden cardiac death	1 (0.1)	0 (0.0)	>0.999
Cancer	124 (16.7)	6 (6.1)	0.006

BMI: body mass index; BUN: blood urea nitrogen; DM: diabetes mellitus; DPP4i: dipeptidyl peptidase-4 inhibitor; SGLT2i: sodium-glucose cotransporter 2 inhibitors; GFR: glomerular filtration rate.

**Table 3:** Comparison between the two groups for cardiovascular disease incidence at 5 years after visiting the emergency room in 2009-2013.

	Total	HYPO_S group	HYPO_M group	*p* value
Ischemic heart disease, *n* (%)	57/374 (15.2)	48/318 (15.1)	9/56 (16.1)	0.851
Cerebrovascular disease, *n* (%)	46/378 (12.2)	39/322 (12.1)	7/56 (13.0)	0.860
Hypertensive disease, *n* (%)	32/385 (8.3)	27/328 (8.2)	5/57 (8.8)	0.799
Aneurysm, *n* (%)	67/411 (16.3)	52/355 (14.7)	15/56 (26.8)	0.022
Heart failure, *n* (%)	26/459 (5.7)	20/389 (5.1)	6/70 (8.7)	0.256
Renal failure, *n* (%)	19/476 (4.0)	14/404 (3.5)	5/72 (6.9)	0.186
Sudden cardiac death, *n* (%)	11/485 (2.3)	9/411 (2.2)	2/74 (2.7)	0.678
Cancer, *n* (%)	30/421 (7.1)	24/350 (6.9)	6/71 (8.5)	0.634

## Data Availability

The data that support the findings of this study are available from the corresponding author upon reasonable request.
